# Extreme intraspecific divergence in mitochondrial haplotypes makes the threespine stickleback fish an emerging evolutionary mutant model for mito-nuclear interactions

**DOI:** 10.3389/fgene.2022.925786

**Published:** 2022-09-08

**Authors:** Emily A. Beck, Susan Bassham, William A. Cresko

**Affiliations:** ^1^ Institute of Ecology and Evolution, University of Oregon, Eugene, OR, United States; ^2^ Presidential Initiative in Data Science, University of Oregon, Eugene, OR, United States

**Keywords:** mitochondrial disease, mitogenome, co-evolution, dysfunction, outbred

## Abstract

Mitochondrial DNA is primarily maternally inherited in most animals and evolves about 10 times faster than biparentally inherited nuclear DNA. Mitochondrial dysfunction (mt-dys) arises when interactions between the co-evolving mitochondrial and nuclear genomes are perturbed in essential processes like oxidative phosphorylation (OXPHOS). Over time mt-dys can lead to mitochondrial diseases (mt-diseases), which are surprisingly prevalent and include common diseases such as Alzheimer’s, Parkinson’s, and diabetes. Unfortunately, the strong impact that intraspecific mitochondrial and nuclear genetic variation has on mt-disease complicates its study and the development of effective treatments. Animal models have advanced our understanding of mt-disease but their relevance to human conditions is often limited by their relatively low nuclear genetic diversity. Many traditional laboratory models also typically have a single mitochondrial haplotype (mitotype), in stark contrast to over 5,000 mitotypes in humans worldwide. The threespine stickleback fish has an evolutionary history that has made it a favorable evolutionary mutant model (EMM) for studying mito-nuclear interactions and possibly mt-diseases. EMMs are species with naturally evolved states that mimic maladaptive human diseases. In threespine stickleback, a period of isolation followed by introgression of the mitochondrial genome from a sister species resulted in the maintenance of two distinct mitochondrial haplotypes which continue to segregate within many populations of wild stickleback. The existence of two mitogenomes segregating in numerous genetically diverse populations provides a unique system for exploring complex mito-nuclear dynamics. Here we provide the first complete coding region analysis of the two threespine stickleback mitotypes, whose mitogenomic divergence exceeds that of other mammalian models for mitochondrial disease and even that between ancient and modern humans. We find that divergence is not uniform across the mitogenome, but primarily impacts protein coding genes, and significantly impacts proteins in Complex I of OXPHOS. The full characterization of these highly divergent intraspecific mitotypes provides a foundation for the development of threespine stickleback as an EMM for mito-nuclear interactions.

## Introduction

Typical metazoan mitochondrial genomes (mitogenomes) are small circular genomes encoding 13 proteins, two rRNAs and 22 tRNAs. Mitogenomes are overwhelmingly maternally inherited in most animals in contrast to the biparentally inherited nuclear genome (Hutchison et al., 1974; Giles et al., 1980), and they evolve rapidly compared to nuclear counterparts due to the less sophisticated prokaryotic DNA repair machinery present in mitochondria (Haag-Liautard et al., 2008; Itsara et al., 2014; Radzvilavicius et al., 2016). Despite these partially independent evolutionary trajectories, components of the mitogenome and nuclear genome must correctly interact to perform essential functions like oxidative phosphorylation (OXPHOS), innate immunity, and regulation of apoptosis (Tait and Green 2010; Cloonan and Choi 2013; Mishra and Chan 2014). Functional incompatibilities between the faster evolving mitogenome and the slower evolving nuclear genome can cause dysfunction (Burton and Barreto 2012; Mishra and Chan 2014; Wolff et al., 2014) which leads to a range of problems including accumulation of mutagenic reactive oxygen species (ROS) and reduction of ATP production, which overtime can lead to mitochondrial disease (mt-disease) (Dautant et al., 2018; Hahn and Zuryn 2019).

Mt-dys and resultant mt-diseases are surprisingly prevalent in humans. Primary mitochondrial diseases (PMD), are defined as diseases caused by a mutation in a protein comprising a subunit of OXPHOS, including diseases such as Leigh’s Syndrome, Pearson Syndrome, and Barth Syndrome. The definition of mt-disease has further expanded to include secondary mitochondrial diseases (SMD). These include any disease exhibiting mt-dys as a symptom, such as Parkinson’s Disease, Alzheimer’s Disease, diabetes, cancer, muscular dystrophy, and amyotrophic lateral sclerosis (ALS) (Guo et al., 2013; Nicholson 2014; Niyazov et al., 2016). The identification of mt-dys as a pervasive issue underlying many common diseases has intensified interest in mt-dys. However, understanding mt-dys disease implications remains complicated by the strong role that mitochondrial and nuclear genetic variation has on symptom severity and treatment efficacy (Benit et al., 2010).

Clinical studies have been extremely valuable in identifying disease-causing genetic variants (Claussnitzer et al., 2020), and model organism research has helped improve mechanistic understanding of disease-causing variants via forward and reverse genetic approaches (Wangler et al., 2017; Cano-Gomez and Trynka 2020; Baldridge et al., 2021). Unfortunately, for several reasons forward- and reverse-genetic screens in model organisms often fail to identify subtle phenotypes that lead to disease later in life (Albertson et al., 2009; Beck et al., 2021) and are unable to fully recapitulate the roles nuclear and mitogenomic variation play in mt-disease (Benit et al., 2010). Most laboratory lines are inbred by design and contain a single mitochondrial haplotype (mitotype), poorly modeling the over 5,000 human mitotypes worldwide (Pipek et al., 2019). An important addition to human and traditional model organism research therefore comes from evolutionary genetics and the use of evolutionary mutant models (EMMs) (Albertson et al., 2009; Beck et al., 2021). EMMs do not exhibit a disease phenotype, but instead have adaptative modifications of homologous gene pathways that would cause disease in humans. EMMs therefore provide models for positive phenotypic outcomes in the context of disease mutations, guiding exploration of genetic compensation and discovery of novel therapeutics (Albertson et al., 2009; Beck et al., 2021). EMMs arise via similar mechanisms as disease mutations in human populations in organisms with diverse genetic backgrounds, making them excellent bridges between human and model organism research (Albertson et al., 2009; Beck et al., 2021).

Despite recent advances using EMMs to better understand several diseases (Albertson et al., 2009; Beck et al., 2021), a missing component has been an EMM with natural mitogenomic and nuclear genetic variation that can be used to understand mito-nuclear dynamics. Threespine stickleback fish (*Gasterosteus aculeatus*) have an evolutionary history that makes them well-suited EMMs for mito-nuclear interactions (Orti et al., 1994; Yamada et al., 2001; Lescak et al., 2014; Ravinet et al., 2018). During the Pleistocene Glacial Maximum, the Sea of Japan was largely enclosed, trapping a subset of *G. aculeatus* from the larger global population spread across the North Arctic (Orti et al., 1994). During a prolonged period of isolation, the trapped population of *G. aculeatus* hybridized with its sister species, *G. nipponicus—*the Japan Sea stickleback. Through hybridization the highly divergent mitochondrial genome from *G. nipponicus* introgressed to *G*. *aculeatus* along with parts of the nuclear genome resulting in a population of *G. aculeatus* hosting a mitogenome now called the Trans-North-Pacific (TNP) mitotype that had accumulated many mutations (Orti et al., 1994; Yamada et al., 2001; Lescak et al., 2014; Ravinet et al., 2018). When glacial ice retreated, the TNP threespine stickleback rejoined the oceanic threespine stickleback where the original Euro-North American (ENA) mitotype was still segregating. This mingling resulted in interbreeding *G. aculeatus* with distinct mitotypes (Orti et al., 1994; Yamada et al., 2001; Lescak et al., 2014; Ravinet et al., 2018).

The existence of two independently evolved mitotypes within a natural population is promising for an EMM of mt-dys on its own. What makes the threespine stickleback system particularly unique is that the segregation of these two distinct mitotypes is happening in many regional freshwater and marine populations in the North Arctic, all with varying ratios of TNP/ENA mitotype individuals (Lescak et al., 2014). As a consequence, there are many natural ‘experiments’ generating countless mito-nuclear genetic combinations in the context of multiple environments. Still, there is little known about the extent of divergence and physiological impact of these mitotypes. Fortunately, threespine stickleback is a genetically amenable organism easily maintained in the laboratory and has been used for decades as a model for population genetics, behavioral studies, and developmental biology (Bell and Foster 1994; Colosimo et al., 2004; Cresko et al., 2004; Cresko et al., 2007; Hohenloe et al., 2010; Kimmel et al., 2012; Miller et al., 2014; Glazer et al., 2015; Greenwood et al., 2015; Peichel and Marques 2017). It is also an emerging model in immunology (Milligan-Mhyre et al., 2016; Small et al., 2017; Weber et al., 2017; Beck et al., 2020; Fuess et al., 2021). A high-quality reference genome is available (Jones et al., 2012; Peichel et al., 2017) and tools for genetic manipulation have been developed, making threespine stickleback an even more promising EMM for disease research.

Until now, the TNP and ENA threespine stickleback mitotypes have been distinguished solely based on divergence of the *cytb* gene for which a restriction digest assay was developed for rapid genotyping of individuals within a population (Orti et al., 1994; Lescak et al., 2014). While whole *Gasterosteus* mitogenomes have previously been assembled for studies of introgression (Ravinet et al., 2018), a complete analysis of the mitogenomes has been lacking. Here we present the first complete assessment of the ENA mitogenome coding sequence. We identify marked levels of nucleotide divergence exceeding that of other vertebrate sister species used to study mito-nuclear discordance, as well as surpassing divergence between modern and ancient humans. Interestingly, the observed divergence is primarily within protein coding genes and is disproportionately present in proteins involved in Complex I of OXPHOS, which could prove to be particularly useful in the study of mitochondrial diseases.

## Materials and methods

### Sample collection and data curation

We assembled mitogenomes for 34 threespine stickleback to be compared to the reference genome (TNP mitotype from Bear Paw Lake, Alaska) from Ensembl; total n = 36 mitogenomes. For population data, we extracted mitochondrial sequences from genomic data on the Sequence Read Archive (SRA) for six TNP fish from the Akkeshi River System in Japan and six ENA fish from Limfjord, Denmark. Accession numbers are summarized in Supplementary Table S1. We also generated new mitochondrial sequencing for 22 fish (ENA mitotype) collected from North America, including one ENA fish from Alaska in a region of TNP/ENA sympatry and for 21 allopatric fish from Oregon (ENA only) with population selection informed by Catchen et al., 2013 and Currey et al., 2019 (Supplementary Table S1). All collections were performed following University of Oregon Institutional Animal Care and Use Committee (IACUC) protocols. Oregon collections were approved under Oregon Department of Fish and Wildlife scientific taking permit numbers 19122 and 20,770. Alaska collections were approved under permit number SF-2011-153. For outgroup species comparisons, we obtained mouse (*Mus musculus* and *M m domesticus*)*,* modern human (*Homo sapiens*)*,* and ancient human (*H. s. denisova*, *H. s. neanderthalensis*, *H. heidelbergensis*) mitogenomes (GenBank numbers DQ874614.2, FJ374649.1, NC_012920.1, KX663333.1, MT677921.1, NC_023100.1 respectively).

### Sequencing library preparation for mitogenomes

We isolated DNA from somatic tissue using standard methods and amplified the mitogenome from these genomic templates via PCR. To do so we designed five primer pairs (Supplementary Table S2) to amplify overlapping fragments that covered the mitogenome and that were optimized for long-range PCR using Phusion High-Fidelity DNA Polymerase. DNA concentrations post-amplification for each fragment were assessed using fluorometry via High Sensitivity Qubit (Supplementary Table S3 **)**. When possible, we standardized DNA to 200 ng and pooled fragments for each sample prior to library preparation. We sheared the pooled amplicons to a range of 300–400 base pairs (bp) using sonication (Covaris, Woburn, United States). Libraries were prepared from these fragments using end repair via NEB Quick blunting reactions, followed by addition of A-overhangs, T4 ligation to uniquely barcoded Illumina-compatible adapters, and total library amplification. We then multiplexed libraries and sequenced on the HiSeq4000 in the UO Genomics and Cell Characterization Core Facility (GC3F) generating paired-end 150 bp reads (Supplementary Table S1).

### Mitogenome assemblies and annotations

We aligned demultiplexed 150 bp reads to the Bear Paw TNP reference mitogenome from Ensembl using the Burrows-Wheeler Aligner (BWA) (Li and Durbin 2009) and filtered using Samtools view (Li et al., 2009) to a minimum MAPQ score of 20. We called consensus sequence on filtered bam files using Geneious (version 10.2.2) and generated a multiple alignment of all consensus sequences in Geneious (version 11.0.12) (https://www.geneious.com) using MUSCLE. We then trimmed the multiple alignment to the limits of the coding region to exclude the partially assembled control region. Therefore, we included annotated 22 tRNAs, two rRNAs, and 13 coding genes based on the reference annotation from Ensembl. Because tRNAs were not specifically annotated in the reference, we confirmed the identity of each tRNA using tRNAscan-SE v2.0 (Chan et al., 2021). For external species comparisons (mouse and primate), we additionally performed multiple alignments within each group in Geneious (version 11.0.12) (https://www.geneious.com) via MUSCLE and annotated genes based on reference annotations from Ensembl.

### Statistical analyses and accounting for heteroplasmy

**FIGURE 1:**
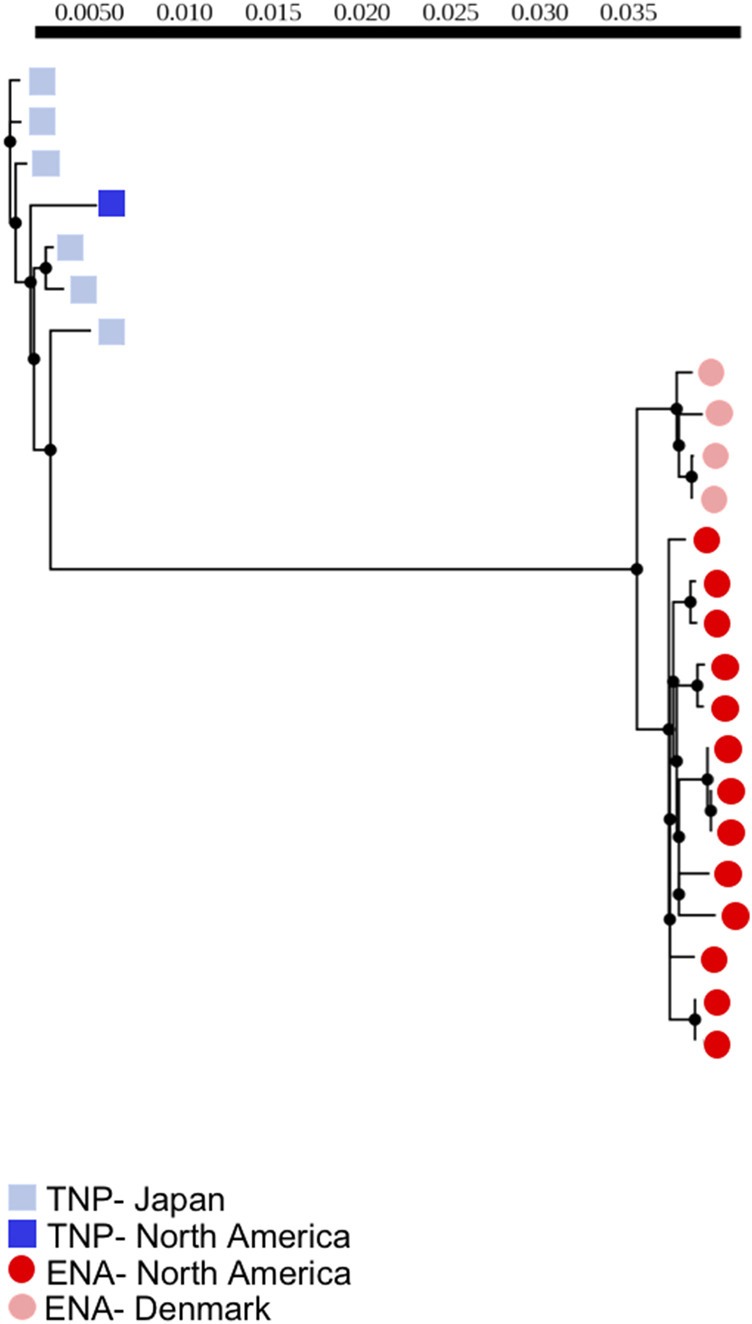
Maximum-likelihood phylogeny of threespine stickleback (*Gasterosteus aculeatus*) whole mitogenomes (excluding the control region) of individuals with no evidence of heteroplasmy. Branch lengths denote nucleotide divergence. Dark red circles indicate fish from North America with the Euro-North American (ENA) mitotype, light red circles indicate fish from Denmark with the ENA mitotype, dark blue squares indicate fish from North American with the Trans-North-Pacific (TNP) mitotype, light blue squares indicate fish from Japan with the TNP mitotype. Scale bar correlates to branch lengths and indicates level of divergence.

We calculated pairwise percent sequence identity using Geneious (version 11.0.12) (https://www.geneious.com) and calculated population genetic statistics using DnaSP v6 (Rozas et al., 2003; Rozas et al., 2017). We also generated a whole mitogenome maximum-likelihood phylogeny using PhyML (Guindon et al., 2010). Due to the presence of heteroplasmy in some samples we had to account for ambiguous sites in our consensus sequences. Because we did not have population-level data for each of our samples we were unable to consistently PHASE genotypes. Instead, we opted for conservative approaches for estimating divergence and generating our phylogeny. For all analyses performed in DnaSP, all SNPs in all samples at locations containing ambiguous sites were removed from analyses, therefore lowering estimates of divergence. In Geneious, sites of heteroplasmy were treated as heterozygous sites using IUPAC standard coding. Percent identity was then adjusted based on the relationship between the heterozygous site and the other alleles to which it was being compared. For example, Y indicating a C/T heterozygous site is considered to have more identity with a C or T than a G or A. For the phylogeny generated in PhyML we also took a conservative approach, with heterozygous sites sharing an allele in the other populations being treated as non-divergent. For example, a Y is considered to be identical to a C or a T. In our phylogenies, we generated maximum-likelihood trees including all individuals (Supplementary Figure S1) and with only individuals showing no evidence of heteroplasmy (Figure 1). Finally, to assess the impact of genetic variants on tRNA structures we used tRNAscan-SE v 2.0 (Chan et al., 2021).

## Results

### Mitogenomic variation exists primarily between mitotypes irrespective of population

We assembled and annotated 34 mitogenomes from 24 populations to be compared to the reference genome (TNP) in order to assess divergence among and within populations, as well as between ENA and TNP mitotypes. These included six genomes from a single population in Denmark (ENA), six genomes from a single population in Japan (TNP), and 22 individuals from different populations in North America (ENA). To standardize comparisons to other models of mitochondrial divergence, we trimmed the assemblies to exclude the highly variable control region and included the full suite of 13 protein coding, two rRNA, and 22 tRNA genes.

We used maximum-likelihood phylogenetic analyses to assess divergence. We found that the majority of mitogenomic divergence was between mitotypes irrespective of geographic origin of population, as indicated by a clear phylogenetic clustering of all ENA mitotype fish to the exclusion of TNP fish with branch lengths between mitotypes greatly exceeding those separating other groups (Figure 1). We additionally identified small levels of within-individual mitogenomic variation but did not identify any individual mitogenome sequence outliers exhibiting extreme patterns of divergence (Figure 1), suggesting that within-individual divergence is low compared to between-mitotype divergence, at least in the context of the single tissue sampled from each fish which prevents us from testing for tissue specific mutations within individuals. We also identified some population-level variation, particularly between allopatric Denmark and North American populations, with Denmark ENA fish clustering separately from North American ENA fish. Importantly, however, we found that the North American TNP fish - from a geographic region of admixture with the ENA mitotype - clustered with the TNP fish from Japan and not ENA North American individuals (Figure 1).

### Mitogenomic variation is consistent with the life history of stickleback

We found that divergence between mitotypes was not due to positive selection by calculating Tajima’s D. We did not identify any significant Tajima’s D values—positive or negative—but did find largely negative Tajima’s D values in the Japan populations consistent with a population expansion (Supplementary Table S4) (Tajima 1989). The finding of no selection is consistent with introgression, and not rapid evolution, as the presence of a second mitotype. Importantly, a population expansion in the TNP population is consistent with the previous hypothesis about the history of these mitotypes because the small population of TNP *G. aculeatus* would have undergone a population expansion at the end of the glacial maximum once they rejoined the Pacific Ocean populations (Orti et al., 1994; Yamada et al., 2001; Lescak et al., 2014; Ravinet et al., 2018).

### Intraspecific stickleback mitotype variation exceeds mitogenomic variation between subspecies of mouse and between modern and ancient humans

We identified rates of intraspecific mitogenomic divergence between threespine stickleback mitotypes that exceeded that of previously identified notable intersubspecific mitogenomic divergence for two sets of subspecies pairs (Table 1). We compared ranges of whole stickleback mitogenome divergence (excluding the highly variable control region) with the sister species pair *Mus musculus* and *M.m. domesticus*, which are used to study mito-nuclear dynamics because of the high mitogenomic divergence between the taxa (Ma et al., 2016). We also made comparisons with the divergence between modern (*Homo sapiens*) and ancient humans (*H.s. neanderthalensis; H.s. denisova*; and *H. heidelbergensis* ). These results confirm that what we document in the single stickleback species is an unusual amount of mitotype divergence, as it exceeds that of mitogenomic divergence between subspecies.

**TABLE 1:** Mitogenomic coding divergence in threespine stickleback (*Gasterosteus aculeatus*) compared to mammalian models for mitochondrial divergence. TNP indicates the Trans-North-Pacific mitotype and ENA indicates the Euro-North American mitotype.

Comparison	Nucleotide divergence
Threespine stickleback (*G. aculeatus*) TNP vs*.* ENA	0.032–0.034
Mouse (*Mus musculus* vs*. M m domesticus*)	0.023
Human (*Homo sapien sapiens* vs*. H. s. neanderthalensis*)	0.011
Human (*Homo sapien sapiens* vs*. H. s. denisova*)	0.02
Human (*Homo sapien sapiens* vs*. H. s. heidelbergensis*)	0.026

### Mitochondrial heteroplasmy detected in some populations of threespine stickleback

In several populations we identified fish that had polymorphic nucleotide sites in their mitogenome assemblies, indicating either paternal leakage of mitochondria or spontaneous mutations that led to more than one mitochondrial haplotype within single individuals. Such a condition is called mitochondrial heteroplasmy (Supplementary Table S5). In all cases, evidence of heteroplasmy was found in allopatric ENA populations and we saw no evidence of both mitotypes present in individuals from regions of mitotype admixture. Unfortunately, given the limited sample size and lack of parentage data from our wild caught fish, we are unable to disentangle paternal leakage from spontaneous convergent mutations, but the abundance of heteroplasmy in specific populations could provide avenues to study genetic mechanisms underlying the maintenance of mitochondrial heteroplasmy.

### Stickleback mitotype divergence lies primarily in protein coding genes

**FIGURE 2:**
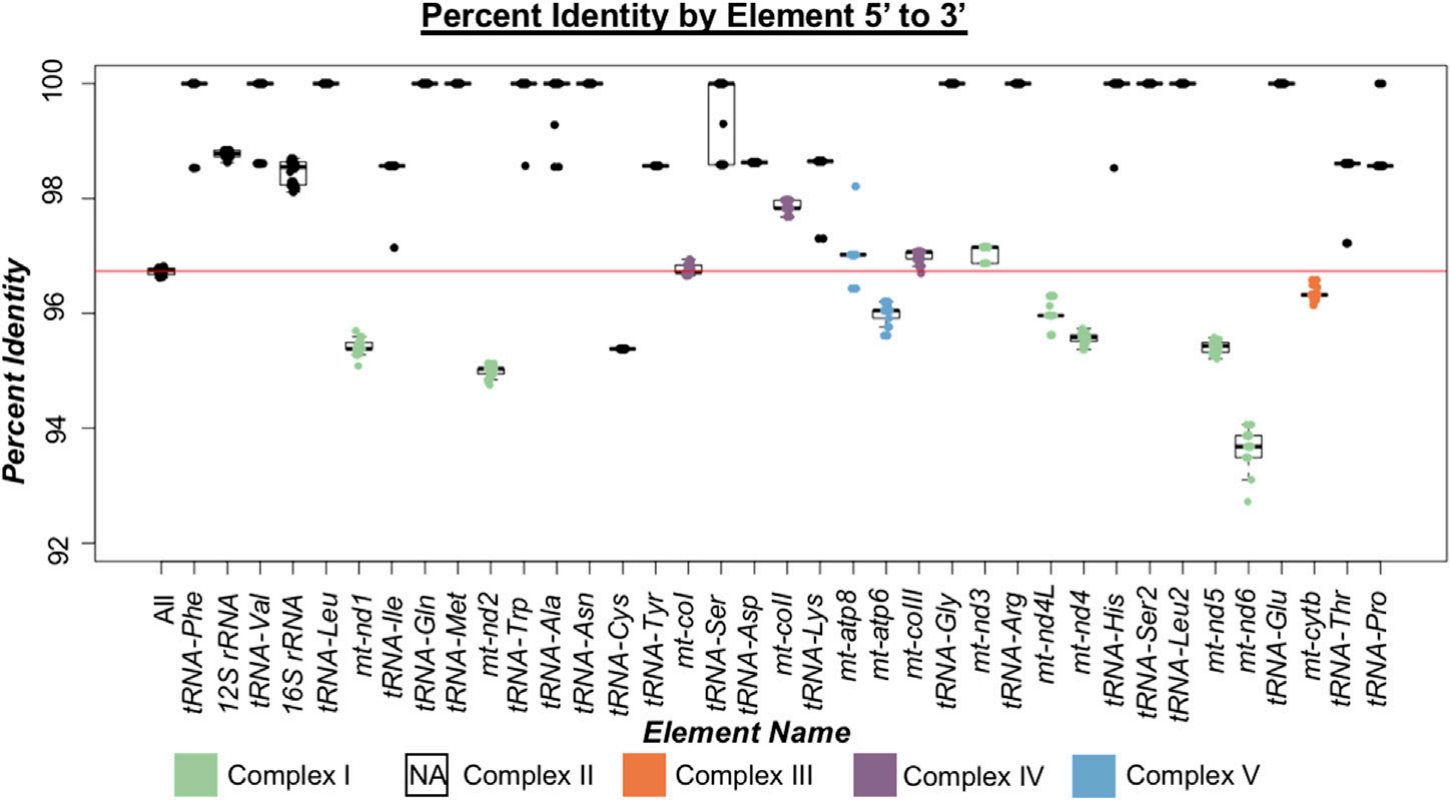
Percent identity of mitogenomic elements 5′ to 3′ in threespine stickleback (*Gasterosteus aculeatus*). Box plots indicate the range of percent identity between the Trans-North-Pacific (TNP) reference genome from North America and Euro-North American (ENA) mitogenomes from North America. The first box plot represents the percent identity across the whole mitogenome (excluding the control region). Box plots for each genic element are ordered based on their 5′ to 3′ orientation. Protein coding genes are colored based on which oxidative phosphorylation (OXPHOS) complex they comprise. Complex II of OXPHOS does not contain mitochondrially encoded proteins and therefore not represented indicated by NA (not applicable). The red horizontal line indicates average mitogenomic percent identity.

By assessing between mitotype divergence in each protein-coding and non-coding gene (tRNAs and rRNAs) through comparisons of North American ENA fish to the North American TNP reference genome (Figure 2), we found that between mitotype divergence is not uniform across the mitogenome. We did not assess intergenic content as mitogenomes contain little intergenic material—65 bp total in stickleback—outside of the excluded control region. Generally, we found that protein-coding gene divergence fell below genome-wide average and non-coding genes trended above average (Figure 2; Tables 2, 3) with a few exceptions: *trna-cys* exhibited below genome-wide average sequence identity and the protein-coding gene mt-*coII* had above average identity. For protein-coding genes there was no correlation between gene size and average between mitotype percent sequence identity (Adjusted *R* ^2^ = -0.052, *p* = 0.53) (Figure 2; Table 2). For non-coding genes there was also no correlation between size and average percent sequence identity (Adjusted *R* ^2^ = -0.006, *p* = 0.36) (Figure 2; Table 3), though it is likely that the observation of low sequence identity in *trna-cys* could be in part because it is the shortest length of any element in the mitogenome and therefore any change would have a proportionally large impact on its percent identity (Table 3; Supplementary Table S6). Analysis of Variance (ANOVA) across genic elements supports significant deviations from genome-wide averages in most elements (Tukey post hoc test summarized in Supplementary Table S7) including strikingly low sequence identity in several protein-coding genes especially mt-*nd6*, which exhibited the lowest sequence identity of any element (Figure 2).

**TABLE 2:** Between mitotype divergence in proteins in threespine stickleback (*Gasterosteus aculeatus*), excluding ambiguous sites.

Protein	Length (bp)^a^	D_N_ ^b^	D_S_ ^c^	P_N_ ^d^	P_S_ ^e^	N.I.^f^	*P*_*Y*ates_ ^g^
ND1	975	1	35	9	28	11.25	0.015
ND2	1047	3	38	10	27	4.69	0.04
ND3	351	0	6	2	6	NA	ns
ND4	1381	3	38	8	34	2.98	ns
ND4L	297	0	8	1	5	NA	ns
ND5	1839	5	58	19	47	4.69	0.004
ND6	522	3	18	2	17	0.71	ns
CytB	1141	1	26	5	28	4.64	ns
COI	1551	0	39	2	41	NA	ns
COII	691	0	12	3	11	NA	ns
COIII	786	0	18	3	19	NA	ns
ATPase6	684	0	17	4	26	NA	ns
ATPase8	168	0	3	2	4	NA	ns

a length in base pairs of the related protein coding gene based on the Trans-North-Pacific (TNP) reference genome;

b number of fixed nonsynonymous changes between mitotypes;

c number of fixed synonymous changes between mitotypes;

d number of polymorphic nonsynonymous changes within Euro-North American (ENA) fish from North America;

e number of polymorphic synonymous changes within ENA, North American fish;

f McDonald-Kreitman Neutrality Index;

g probability with Yates’ correction. Horizontal lines denote protein groups by OXPHOS, Complex (I, III, IV, and V respectively).

**TABLE 3:** Polymorphisms in non-coding mitogenomic elements between and within mitotype in threespine stickleback (*Gasterosteus aculeatus*).

Gene	Length (bp)^a^	P_TNP_ ^b^	P_ENA_ ^c^	P_shared_ ^d^	F^e^
*12S-rRNA*	946	5	6	0	9
*16S-rRNA*	1690	8	29	1	15
*tRNA-asp*	73	1	0	0	0
*tRNA-cys*	65	1	1	0	1
*tRNA-his*	68	0	1	0	0
*tRNA-ile*	70	1	1	0	1
*tRNA-lys*	74	2	1	0	0
*tRNA-phe*	68	0	2	0	0
*tRNA-pro*	70	0	1	0	0
tRNA-ser-2*	68	1	0	0	0
*tRNA-thr*	72	0	2	0	1
*tRNA-trp*	70	0	1	0	0
*tRNA-tyr*	70	1	0	0	0
*tRNA-val*	72	0	1	0	0

Asterisk after number 2 indicates it is the 3’ copy of *tRNA-Ser*.

a length in base pairs based on the Trans-North-Pacific (TNP) reference genome;

_b_ polymorphisms exclusive to TNP mitotype;

c polymorphisms exclusive to the Euro-North American (ENA) mitotype;

d polymorphisms shared between TNP and ENA mitotypes;

e fixed differences between TNP and ENA mitotypes.

### Protein coding divergence is primarily in complex I of OXPHOS

**FIGURE 3:**
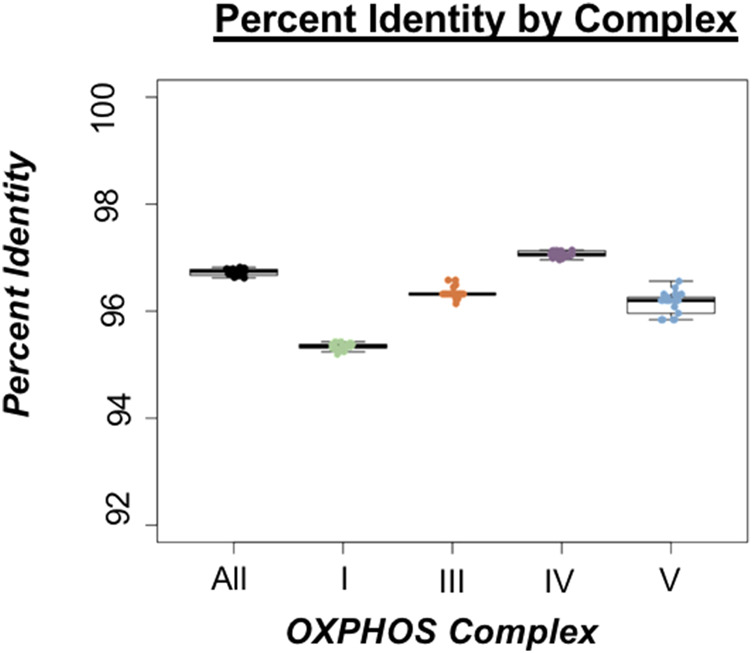
Percent identity of oxidative phosphorylation (OXPHOS) complexes in threespine stickleback (*Gasterosteus aculeatus*). Box plots indicate the spread of percent identity between the Trans-North-Pacific (TNP) reference genome from North America and Euro-North American (ENA) mitogenomes from North America. Color choices are consistent throughout the manuscript.

We observed the highest rates of divergence in proteins involved in Complex I of OXPHOS comprised of ND1, ND2, ND3, ND4, ND4L, ND5, and ND6 (Figure 2) suggesting sequence divergence correlates with some aspect of protein function. ANOVA indicated significant deviations from genome wide average across all OXPHOS complexes, but Complex I exhibited the most divergence (Figure 3; Supplementary Table S8). This result was not surprising given that five of the seven proteins comprising Complex I exhibited the lowest sequence identity of any of the coding genes (ND1, ND2, ND4, ND5, and ND6). Complex II does not contain any mitochondrially encoded proteins and was therefore excluded from analysis. The single Complex III mitochondrial protein exhibited slightly below average sequence identity (Figure 3), while proteins from Complexes IV and V showed a wide range of values both above and below genome wide averages of sequence identity (Figures 2, 3). Combined, these data reveal a pattern of increased divergence in proteins functioning early in the OXPHOS Pathway.

We found evidence of strong purifying selection in some protein-coding genes when we combined all TNP samples from Japan and North America to compare with the North American ENA sample. Using a McDonald-Kreitman 2x2 test for selection (McDonald and Kreitman 1991), we assessed rates of synonymous and nonsynonymous polymorphisms within each population, and synonymous and nonsynonymous fixed differences between mitotypes. We observed an increase in synonymous changes compared to nonsynonymous changes indicating significant purifying selection (Table 2) and found no evidence of positive selection. A lack of positive selection was expected due to the introgression of the mitogenome (Orti et al., 1994; Yamada et al., 2001; Lescak et al., 2014; Ravinet et al., 2018), and strong purifying selection was expected based on previous evidence of consistently strong purifying selection on mitogenomic mutations (Stewart et al., 2008). There were, however, fixed nonsynonymous sites only in proteins from Complexes I and III as well as higher levels of nonsynonymous polymorphisms in these two complexes compared to late acting complexes IV and V, again supporting increased divergence early in OXPHOS (Table 2).

## Discussion

Existing models for mt-dys and mt-disease lack some desirable attributes, such as natural genetic variation in nuclear and mitochondrial genomes. This deficit has hindered the study of mt-dys, which has been repeatedly shown to be heavily influenced by both mitochondrial, nuclear, and gene-by-environment variation (Benit et al., 2010; Zanon et al., 2018). There are important examples of hybridizable pairs of taxa with mitochondrial variation, including murine models *M m musculus* and *M.m domesticus* (Ma et al., 2016) and mummichog fish subspecies *Fundulus heteroclitus* and *F. heteroclitus macrolepidotus* (Flight et al., 2011; Baris et al., 2017)*.* These existing outbred models, however, are commonly studied in single populations or at discrete, rare hybrid zones. What has been needed is a widespread, outbred model for mito-nuclear dynamics maintaining mitogenomic variation in multiple environments. Additional animal models exhibit mitochondrial-nuclear introgression with the mitogenome of the species with the larger effective population size introgressing and replacing that of the smaller effective population size species. A good example is the laboratory amenable system *Drosophila yakuba* and *D. santomea* (Llopart et al., 2005; Llopart et al., 2014; Beck et al., 2015) in which the *D. santomea* mitogenome was replaced by *D. yakuba.* In this case, no mitotype admixture is possible as only the *D. yakuba* mitotype exists. One possible explanation for the maintenance of divergence and admixture in the threespine stickleback system is the large global effective population size and widely dispersed populations of *G. aculeatus* (originally ENA mitotype) throughout the Northern Hemisphere. As such, there has not been enough time for the *G. nipponicus* TNP-mitogenome to replace the endogenous ENA-mitogenome (Ravinet et al., 2018). We are therefore studying this system at a very fortuitous point in evolutionary time where studies of mito-nuclear interactions can occur during a window of admixture.

Our quantification of intraspecific mitotype variation in threespine stickleback makes a strong case for its use as an outbred model for mito-nuclear interactions. First, mitogenomic variation in threespine stickleback is primarily between two highly divergent mitotypes (TNP and ENA) rather than among populations or within individuals. Second, the observed level of divergence also exceeds that of existing outbred models with whole mitogenome assemblies including *M m musculus* and *M.m domesticus* and exceeds that of modern and ancient humans. Third, hybridization and admixture of stickleback mitotypes has occurred repeatedly in many different freshwater and oceanic environments, generating a multitude of natural experiments for the study of mito-nuclear dynamics in the context of environmental variation.

These strengths are extremely valuable in addressing questions concerning mito-nuclear dynamics in the context of environmental and genetic heterogeneity. Fortunately, while threespine stickleback are a promising EMM due to their repeated mitotype admixture in nature, they are also amenable to laboratory studies. As in zebrafish, developmental and physiological studies are facilitated by external fertilization in stickleback (Bell and Foster 1994). Threespine stickleback can also be crossed and reared for QTL/eQTL mapping studies (Greenwood et al., 2015; Peichel and Marques 2017; Beck et al., 2020), and they can be genetically manipulated using technology like CRISPR-Cas9 genome editing (Wucherpfennig et al., 2019; Xi et al., 2019; Kingman et al., 2021). Recent advances in mitogenome editing could theoretically be applied in this system to further enhance stickleback as an EMM for mitochondrial physiology (Hussain et al., 2021).

Another strength of stickleback as an EMM is the heterogeneity of divergence across the stickleback mitogenome. We observed divergence primarily in coding genes irrespective of their physical location in the mitogenome, with *mt-nd6* being a clear outlier for elevated levels of nucleotide divergence. Importantly, we discovered that much of the protein divergence is in those functioning early in OXPHOS in Complex I, even though Complex I proteins are spread throughout the mitogenome. More work is needed to understand what, if any, are the physiological consequences that exist because of divergence in Complex I proteins within populations. However, these findings are suggestive and intriguing, as Complex I is the primary producer of ROS, which, if not cleared from mitochondria by antioxidants and other proteins in Complex I, can lead to mt-dys and cellular damage (Hirst et al., 2008). As the largest complex in OXPHOS, Complex I presents a large target for mutation and is the source of many disease variants in humans (Carroll et al., 2003; Koopman et al., 2012; Barshad et al., 2019). Divergence in specific regions of the mitogenome is something that remains unexplored in many natural systems, in which studies have primarily used restriction digestion assays or targeted identification of SNPs to characterize mitotypes. Until now, threespine stickleback researchers have relied on a restriction digest to identify SNPs in a PCR amplicon within *mt-cytb* (Orti et al., 1994; Lescak et al., 2014). From our analyses we can conclude that *mt-cytb* was a serendipitous choice as a representative of average mitotype divergence, but it missed the more extreme divergence identified in other areas of the mitogenome. We propose that full mitogenome sequencing is therefore required for the advancement of EMMs for mito-nuclear interactions.

## Data Availability

The data presented in the study are deposited in the Sequencing Read Archive (SRA) repository, accession numbers SAMN30467736 -SAMN30467757.
