# Supplementary figures and images for: Extreme intraspecific divergence in mitochondrial haplotypes makes the threespine stickleback fish an emerging evolutionary mutant model for mito-nuclear interactions
Source: Front Genet. 2022 Sep 8;13:925786. doi: 10.3389/fgene.2022.925786 (PMC9499175; doi:10.3389/fgene.2022.925786)

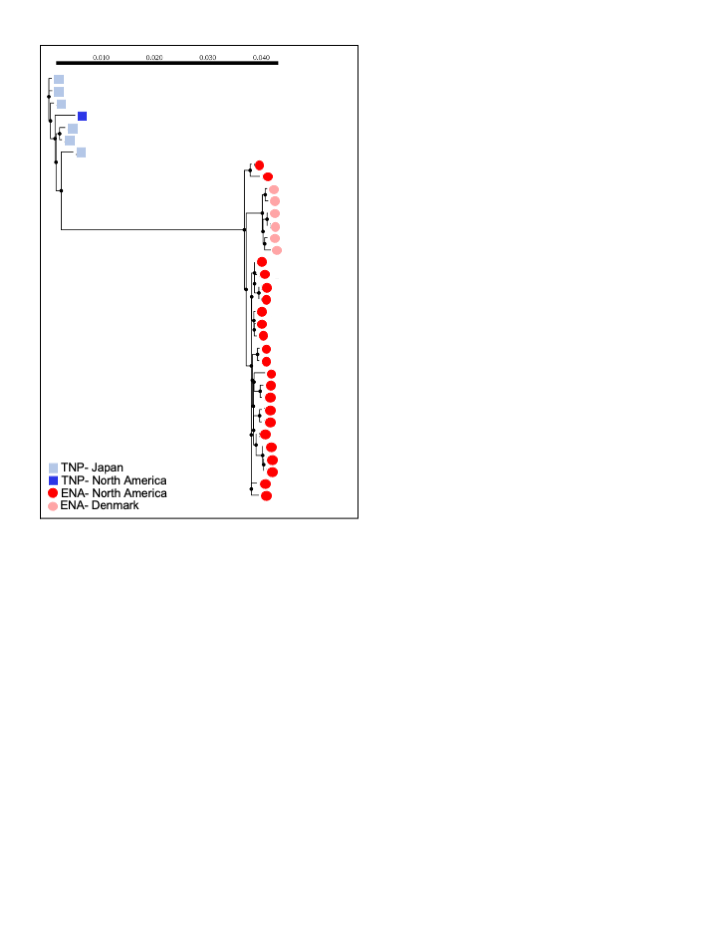

Supplement: Supplementary file 2 [file Image1.TIFF]
