# Supplementary material for: Extreme intraspecific divergence in mitochondrial haplotypes makes the threespine stickleback fish an emerging evolutionary mutant model for mito-nuclear interactions
Source: Front Genet. 2022 Sep 8;13:925786. doi: 10.3389/fgene.2022.925786 (PMC9499175; doi:10.3389/fgene.2022.925786)
Supplement: Supplementary file 4 [file Table9.DOCX]

*Contribution to the Field*

Mitochondrial diseases are characterized by poorly functioning mitochondria largely due to mutations in either the mitochondrial or nuclear genomes. Unfortunately, mitochondrial diseases are difficult to study in inbred models because of the large role genetic variation plays on etiology of mitochondrial dysfunction. What has been needed is an outbred, laboratory-amenable model with mitogenomic variation that allows for studies of mito-nuclear interactions. In this paper we present threespine stickleback fish as a potential model to study mito-nuclear interactions. The threespine stickleback has evolved two distinct mitochondrial haplotypes with divergence that exceeds that of common models for mitochondrial disease. Fish with each mitotype exist in multiple regions of admixture providing natural laboratories to study mito-nuclear combinations and can help elucidate mechanisms for nuclear compensation of mitogenomic variation. Importantly, stickleback are also amenable to genetic manipulations and can be housed in a laboratory making them ideal bridges between human and inbred animal-model studies. Here we provide a full characterization of these two stickleback mitotypes from around the world. We identify regions of high divergence including protein coding genes in Complex I of Oxidative Phosphorylation and make a case for the utility of this unique fish to enhance studies of mitochondrial disease.
